# Real-World Outcomes of Combined Carbon-Ion Radiotherapy and Systemic Immunotherapy for Hepatocellular Carcinoma (Atezolizumab Plus Bevacizumab or Durvalumab Plus Tremelimumab): A Single-Center Retrospective Study

**DOI:** 10.3390/jcm15145449

**Published:** 2026-07-12

**Authors:** Keita Maki, Hiroaki Haga, Takashi Kaneko, Kyoko Hoshikawa, Tomohiro Katsumi, Fumiya Suzuki, Fumi Uchiyama, Takumi Hanatani, Yasuhito Hagiwara, Masashi Koto, Yoshiyuki Ueno

**Affiliations:** 1 Department of Gastroenterology, Faculty of Medicine, Yamagata University, Yamagata City 990-9585, Yamagata, Japan; 2 Department of Radiation Oncology, Faculty of Medicine, Yamagata University, Yamagata City 990-9585, Yamagata, Japan

**Keywords:** hepatocellular carcinoma, immune checkpoint inhibitors, carbon-ion radiotherapy, systemic immunotherapy, atezolizumab plus bevacizumab, durvalumab plus tremelimumab

## Abstract

**Background:** Carbon-ion radiotherapy (CIRT) is an effective local treatment for hepatocellular carcinoma (HCC); however, evidence on systemic immunotherapy after CIRT and the role of CIRT following immunotherapy remains limited. This study aimed to assess clinical outcomes and sequencing of CIRT and systemic immunotherapy in real-world practice. **Methods:** We retrospectively analyzed 41 patients with HCC who received CIRT and systemic immunotherapy (atezolizumab plus bevacizumab or durvalumab plus tremelimumab). Patients were categorized into three groups: (i) systemic immunotherapy for recurrent HCC after CIRT (*n* = 20), (ii) curative-intent CIRT after systemic immunotherapy (*n* = 10), and (iii) CIRT for residual or insufficiently responding lesions (≤3 lesions) after systemic immunotherapy (*n* = 11). **Results:** In group (i), 14 of 20 patients achieved complete or partial responses. These responders had low tumor burden (within the up-to-7 criteria) and preserved liver function (modified albumin–bilirubin grade 1/2a) at treatment initiation and demonstrated significantly longer progression-free and overall survival than those with stable or progressive disease. In group (ii), 4 of 10 patients achieved a clinical complete response and became drug-free; notably, all had solitary intrahepatic tumors at immunotherapy initiation. In group (iii), the rate of transition to clinical complete response was low, and disease control remained limited. **Conclusions:** Systemic immunotherapy demonstrated high effectiveness in recurrent HCC following CIRT, particularly in patients with low tumor burden and preserved liver function. Moreover, curative-intent CIRT after immunotherapy may benefit patients with solitary intrahepatic tumors but appears limited in those with insufficient response to immunotherapy, including residual oligolesions.

## 1. Introduction

Hepatocellular carcinoma (HCC) has a high global incidence and mortality rate and remains one of the leading causes of cancer-related death worldwide [1]. It commonly develops in the setting of chronic hepatitis or liver cirrhosis, and many patients are diagnosed at an advanced stage. Consequently, HCC continues to be associated with a poor prognosis [2]. Moreover, despite advances in locoregional and systemic therapies, HCC is characterized by a high recurrence rate [3,4,5]. In recent years, multidisciplinary treatment strategies combining locoregional therapies—such as radiofrequency ablation, transarterial chemoembolization, radiotherapy, and surgical resection—with systemic immunotherapy have become increasingly common, and further improvements in clinical outcomes are anticipated [6,7,8].

Carbon-ion radiotherapy (CIRT) has attracted attention as an effective local treatment for HCC because of its favorable dose distribution and high relative biological effectiveness [9]. Given its minimally invasive nature, CIRT can be safely applied to older adults and patients with comorbidities and serves as an important curative treatment option [10]. A retrospective cohort study evaluating adverse events (AEs) after CIRT reported that most toxicities were grade 1 or 2 [11], supporting its favorable safety profile.

In real-world clinical practice, CIRT is increasingly incorporated into treatment strategies across multiple clinical settings. In selected patients who achieve tumor downstaging with systemic immunotherapy, curative-intent approaches include CIRT alone or in combination with other local therapies. Accordingly, the clinical role of CIRT continues to expand.

However, although CIRT provides excellent local control, some patients experience intrahepatic distant recurrence outside the irradiated field [12,13]. In such cases, systemic immunotherapy may be initiated after recurrence; however, evidence regarding response rates and overall clinical utility remains limited. Furthermore, the rate of clinical complete response (CR) after curative-intent CIRT following systemic immunotherapy remains inadequately investigated.

In this study, we retrospectively analyzed real-world data from a single institution involving patients with HCC treated with a combination of CIRT and systemic immunotherapy to evaluate treatment outcomes and longitudinal clinical courses in routine clinical practice. In addition, by visually presenting the long-term treatment course, we evaluated the clinical relevance of integrating CIRT with systemic immunotherapy in HCC treatment strategies.

## 2. Materials and Methods

### 2.1. Patient Population

**Figure 1:**
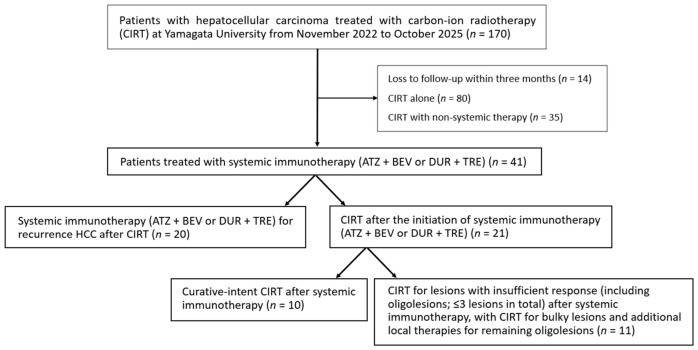
Flowchart of patient selection for HCC treated with CIRT in combination with systemic immunotherapy. ATZ + BEV, atezolizumab plus bevacizumab; DUR + TRE, durvalumab plus tremelimumab; CIRT, carbon-ion radiotherapy; HCC, hepatocellular carcinoma.

In this retrospective single-center study, we reviewed 170 consecutive patients who underwent CIRT at our institution between November 2022—when CIRT became covered by the National Health Insurance system in Japan—and October 2025 (Figure 1). Of these, 129 patients were excluded, including those with a follow-up period of less than 3 months (*n* = 14), those treated with CIRT alone (*n* = 80), and those treated with CIRT in combination with local therapy (non-systemic therapy) (*n* = 35). Consequently, 41 patients who received CIRT in combination with systemic immunotherapy were included in the final analysis.

**Figure 2:**
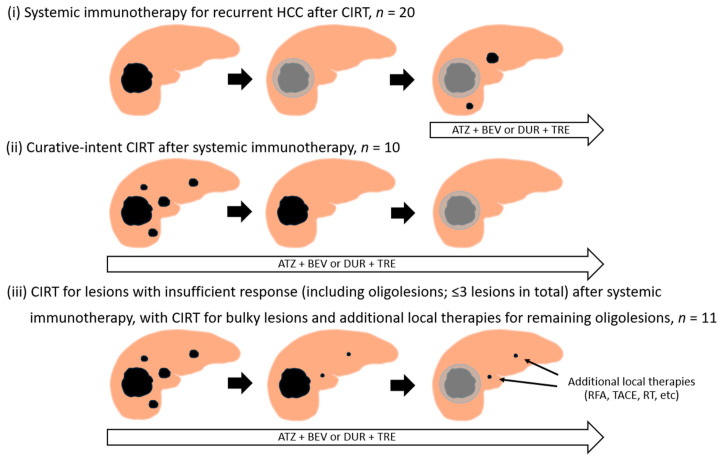
Classification of patients with HCC treated with CIRT in combination with systemic immunotherapy (ATZ + BEV or DUR + TRE) into three groups: (**i**) systemic immunotherapy for recurrence after CIRT, (**ii**) curative-intent CIRT after systemic immunotherapy, and (**iii**) CIRT for lesions with insufficient response (including oligolesions, with a total of ≤3 lesions) after systemic immunotherapy, along with CIRT for bulky lesions and additional local therapies for the remaining lesions or oligolesions. The gray shaded area indicates the irradiation field of CIRT. ATZ + BEV, atezolizumab plus bevacizumab; DUR + TRE, durvalumab plus tremelimumab; CIRT, carbon-ion radiotherapy; HCC, hepatocellular carcinoma; RFA, radiofrequency ablation; RT, radiotherapy; TACE, transarterial chemoembolization.

The latter group was further subdivided, resulting in three final groups: (i) systemic immunotherapy—atezolizumab plus bevacizumab (ATZ + BEV) or durvalumab plus tremelimumab (DUR + TRE)—administered for recurrent HCC following CIRT (*n* = 20); (ii) curative-intent CIRT after systemic immunotherapy in patients whose tumor burden had decreased sufficiently to allow all remaining intrahepatic lesions to be treated with CIRT alone (*n* = 10); and (iii) curative-intent CIRT for lesions with insufficient response (including oligolesions; ≤3 lesions in total) to systemic immunotherapy, in whom CIRT alone was considered insufficient to achieve complete local control and additional local therapies (e.g., radiofrequency ablation [RFA], transarterial chemoembolization [TACE], or radiotherapy) were required (*n* = 11) (Figure 1 and Figure 2). Curative-intent CIRT was defined as treatment delivered with the objective of achieving complete eradication of all radiologically detectable disease. This objective was achieved either by CIRT alone (group ii) or by CIRT combined with planned additional local therapies for residual oligolesions (group iii), provided that no uncontrolled extrahepatic disease was present at the time of treatment planning.

This classification was developed retrospectively after review of clinical courses, in order to delineate the principal clinical scenarios in which CIRT and systemic immunotherapy were integrated in routine practice.

The diagnosis of HCC, as well as the presence of vascular invasion, lymph node metastasis, and distant metastasis, was assessed using contrast-enhanced computed tomography (CT) and/or magnetic resonance imaging (MRI). When HCC could not be definitively diagnosed by imaging, histological confirmation was obtained through tumor biopsy.

### 2.2. Carbon-Ion Radiotherapy

CIRT was delivered using a spot-scanning method with a rotating gantry beam system, enabling 360° irradiation from any angle. Before treatment planning, fiducial metallic markers were implanted near the tumor to ensure accurate positioning during irradiation. To maintain target reproducibility, a low-temperature thermoplastic sheet, a vacuum bag, and a respiratory-gated irradiation system were used during CT-based radiotherapy planning. Gross tumor volume (GTV) was determined using dynamic contrast-enhanced CT and/or MRI. The clinical target volume was generated by adding a 7-mm margin to the GTV. Treatment planning was conducted using a CT-based three-dimensional planning system (RayStation; RaySearch Laboratories, Stockholm, Sweden) and optimized with robustness analysis tools. Setup uncertainties were accounted for by applying a 2-mm margin in the left–right, anterior–posterior, and superior–inferior directions. A 2% range uncertainty parameter was applied in the planning software.

Treatment planning CT images were acquired using four-dimensional CT synchronized with respiratory motion. The images were divided into 10 respiratory phases at 10% increments from 0% to 100%, corresponding to inhalation (0%) and exhalation (50%). Tumor delineation was performed on the 50% (exhalation) phase image. The internal target volume was defined by combining CT images across the maximal respiratory phase range in which GTV motion remained within 3 mm. Only respiratory phases within this range were used during actual irradiation.

Before each treatment session, orthogonal fluoroscopy and radiography were used to confirm and, when necessary, correct the radiation field. The prescribed dose of CIRT was expressed in gray (Gy) and calculated as the absorbed dose multiplied by the relative biological effectiveness of carbon ions. According to the institutional protocol, the prescribed dose for HCC was 60 Gy delivered in four fractions. Treatment was administered once daily, with four fractions delivered on separate days within one week. For organ-at-risk constraints, the remnant liver volume receiving less than 30 Gy for the liver parenchyma was required to be at least 500 cm^3^. In addition, the dose to the most exposed 2 cm^3^ of the digestive tract (D2cc) had to be less than 30 Gy.

### 2.3. Clinical Outcome Evaluation

Patients were evaluated 1 month after CIRT and subsequently every 3 months during the first 2 years, every 6 months during the following 2 years, and annually thereafter. Follow-up assessments included physical examination, laboratory testing, and dynamic contrast-enhanced CT or MRI. Imaging studies were generally performed at 3-month intervals. The last follow-up date was January 2026.

Recurrence after CIRT was defined based on imaging evidence of tumor enlargement or the appearance of newly developed arterial-phase hyperenhancing lesions within the irradiated field at sites where enhancement had previously resolved. Recurrence was assessed using imaging criteria consistent with the modified Response Evaluation Criteria in Solid Tumors (mRECIST).

Progression-free survival (PFS) was defined as the interval from the initiation of systemic immunotherapy to the first occurrence of intrahepatic or extrahepatic recurrence or death from any cause. Overall survival (OS) was defined as the interval from the initiation of systemic immunotherapy to death from any cause.

Tumor response was evaluated according to mRECIST based on a consensus assessment by experienced radiologists. According to mRECIST, CR was defined as the disappearance of intratumoral arterial enhancement in all target lesions, partial response (PR) as a decrease of at least 30% in the sum of diameters of viable (enhancing) target lesions, progressive disease (PD) as an increase of at least 20%, and stable disease (SD) as a disease that did not meet the criteria for PR or PD. The best overall response was defined as the maximum response observed before confirmation of disease progression.

Clinical CR was defined, with reference to a previous report [14], as fulfillment of both of the following criteria: (1) achievement of CR on imaging evaluation using CT or MRI according to mRECIST and (2) normalization of both tumor markers, α-fetoprotein (AFP) and des-γ-carboxy prothrombin (DCP).

Drug-free status was defined as the discontinuation of systemic therapy after confirmation that clinical CR had been maintained for at least 24 weeks.

Toxicity related to CIRT and systemic immunotherapy was assessed using the Common Terminology Criteria for Adverse Events (CTCAE) version 5.0. CTCAE employs a grading scale from 1 to 5 to classify the severity of AEs, where grade 1 denotes mild, grade 2 moderate, grade 3 severe, grade 4 life-threatening, and grade 5 death. Tumor progression and symptoms attributable to the tumor itself were not considered in the evaluation of treatment-related AEs in this study.

### 2.4. Statistical Analysis

PFS and OS in the CR/PR and SD/PD groups were analyzed using the Kaplan–Meier method. Confidence intervals (CIs) were calculated using Greenwood’s formula, and survival curves were compared using the log-rank test.

For continuous variables, normality was assessed using the Shapiro–Wilk test. Normally distributed variables were compared using Student’s *t*-test, whereas nonnormally distributed variables were compared using the Mann–Whitney *U* test. Categorical variables were compared using the chi-square test or Fisher’s exact test, as appropriate, based on expected cell counts.

All statistical analyses were conducted using GraphPad Prism version 10 (GraphPad Software, San Diego, CA, USA). Statistical significance was defined as a two-sided *p*-value < 0.05.

### 2.5. Ethics Approval

This retrospective study was approved by the Ethics Review Committee of Yamagata University School of Medicine (approval no. 2023-110).

## 3. Results

### 3.1. Efficacy of Systemic Immunotherapy for Recurrent HCC After CIRT (Figure 3)

**Figure 3:**
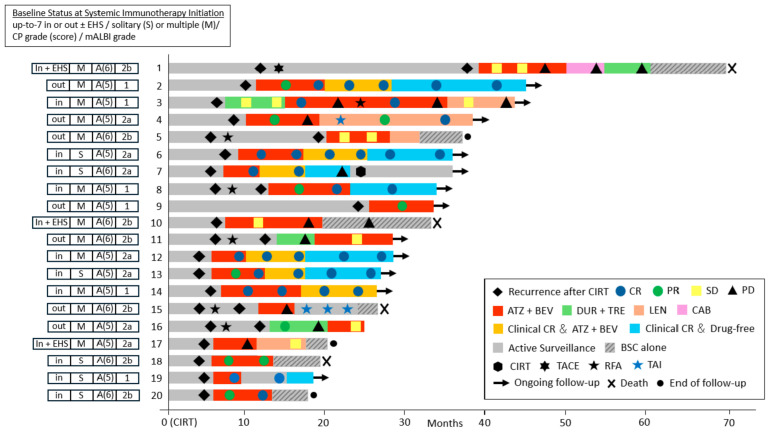
Swimmer plot illustrating treatment duration and best overall response in patients who received systemic immunotherapy for recurrent HCC after CIRT. ATZ + BEV, atezolizumab plus bevacizumab; DUR + TRE, durvalumab plus tremelimumab; LEN, lenvatinib; CAB, cabozantinib; CIRT, carbon-ion radiotherapy; EHS, extrahepatic spread; HCC, hepatocellular carcinoma; CR, complete response; PR, partial response; SD, stable disease; PD, progressive disease; TACE, transarterial chemoembolization; RFA, radiofrequency ablation; TAI, transarterial infusion.

Figure 3 presents a swimmer plot illustrating treatment duration and the best overall response among 20 patients who received systemic immunotherapy for recurrent HCC after CIRT. First-line systemic immunotherapy consisted of ATZ + BEV in 17 patients and DUR + TRE in 3 patients. Of the 20 patients, 14 achieved a best overall response of CR or PR. Among these responders, 8 achieved clinical CR, and 7 subsequently attained drug-free status. The objective response rate (ORR) was 70%, and the disease control rate (DCR) was 90%.

Patients with SD or PD as the best overall response predominantly exhibited tumor burden patterns at recurrence that were either beyond the up-to-7 criteria (up-to-7 out) or included extrahepatic spread (EHS). In contrast, among the 8 patients who achieved clinical CR or clinical CR followed by drug-free status, 7 demonstrated a tumor burden pattern within the up-to-7 criteria (up-to-7 in) at recurrence.

Among the 7 patients who died or reached the end of follow-up, Cases 18 and 20 died or discontinued follow-up because of deterioration in liver reserve, despite having achieved CR or PR as the best overall response.

### 3.2. Longitudinal Changes in Treatment Response Among Patients Receiving Systemic Immunotherapy for Recurrent HCC After CIRT

**Figure 4:**
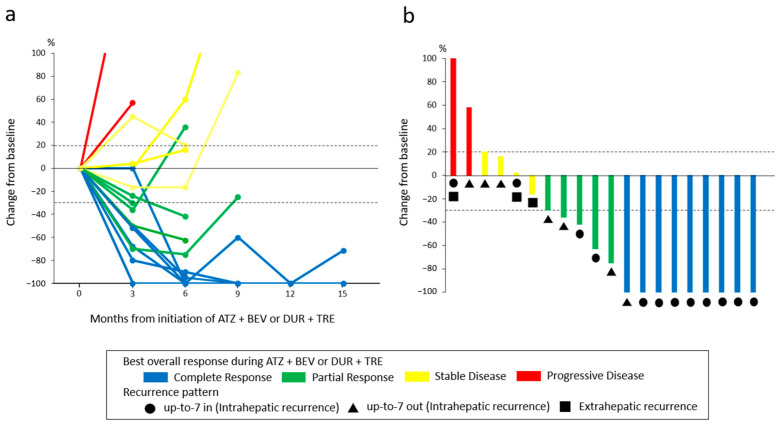
Longitudinal changes in treatment response among patients who received systemic immunotherapy for recurrent HCC after CIRT. (**a**) Spider plot showing changes in tumor diameter following initiation of systemic immunotherapy. (**b**) Waterfall plot showing maximum tumor shrinkage rate and recurrence pattern/tumor distribution in patients treated with systemic immunotherapy for recurrence after CIRT. ATZ + BEV, atezolizumab plus bevacizumab; DUR + TRE, durvalumab plus tremelimumab; CIRT, carbon-ion radiotherapy; HCC, hepatocellular carcinoma.

Figure 4a shows a spider plot illustrating changes in tumor diameter following initiation of systemic immunotherapy (ATZ + BEV or DUR + TRE) for recurrent HCC after CIRT. Overall, many patients demonstrated early tumor shrinkage after treatment initiation. Among patients who achieved CR or PR as the best overall response, sustained reductions in tumor diameter were observed over time. In contrast, many patients with SD as the best overall response subsequently progressed to PD after a defined interval, whereas patients with PD demonstrated early tumor growth.

### 3.3. Association Between Best Overall Response and Recurrence Pattern or Tumor Distribution in Patients Receiving Systemic Immunotherapy for Recurrent HCC After CIRT

Figure 4b presents a waterfall plot illustrating the maximum tumor shrinkage rate after systemic immunotherapy for recurrent HCC following CIRT. Patients who achieved CR or PR as the best overall response exhibited greater tumor shrinkage, whereas those with SD showed limited tumor reduction and those with PD demonstrated tumor growth.

Analysis of the recurrence patterns and tumor distribution in relation to the best overall response revealed that most CR/PR cases had tumor burdens within the up-to-7 criteria at the initiation of systemic immunotherapy for recurrence. In contrast, SD/PD cases were characterized by tumor burdens exceeding the up-to-7 criteria or by extrahepatic spread at recurrence. These findings suggest that the recurrence pattern and tumor distribution at recurrence may influence responsiveness to systemic immunotherapy in HCC.

### 3.4. Comparison Between the CR/PR and SD/PD Groups Among Patients Receiving Systemic Immunotherapy for Recurrent HCC After CIRT

Among the 20 patients who received systemic immunotherapy for recurrent HCC after CIRT, 14 were categorized into the CR/PR group and 6 into the SD/PD group based on best overall response (Table 1). Clinical and tumor-related variables were compared between the two groups at the time of CIRT and at the initiation of systemic immunotherapy: Child–Pugh score, modified albumin–bilirubin (mALBI) grade, up-to-7 criteria (in or out), macrovascular invasion, EHS, AFP, and DCP (Table 1). The CR/PR group had a significantly higher proportion of patients with preserved liver function, defined as mALBI grade 1/2a, at the initiation of systemic immunotherapy (*p* = 0.0076) and included no patients with EHS (*p* = 0.0175).

**Figure 5:**
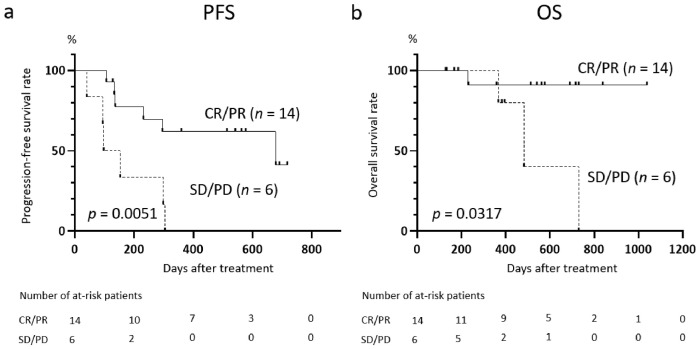
Comparison of PFS and OS between patients with CR/PR and SD/PD after systemic immunotherapy for recurrence following CIRT. (**a**) PFS. (**b**) OS. CIRT, carbon-ion radiotherapy; CR, complete response; OS, overall survival; PD, progressive disease; PFS, progression-free survival; PR, partial response; SD, stable disease.

In addition, the CR/PR group demonstrated significantly longer PFS and OS than the SD/PD group. The 6- and 12-month PFS rates were 77.4% (95% CI, 54.8–100%) and 61.9% (95% CI, 35.6–88.3%), respectively, in the CR/PR group, compared with 33.3% (95% CI, 0–71.1%) and 0% in the SD/PD group (*p* = 0.0051). Median PFS was 678 days in the CR/PR group and 97 days in the SD/PD group (Figure 5a). The 12- and 24-month OS rates were both 90.9% (95% CI, 73.9–100%) and 90.9% (95% CI, 73.9–100%), respectively, in the CR/PR group. In contrast, OS rates in the SD/PD group were 100% at 12 months and 0% at 24 months (*p* = 0.0317). Median OS was not reached in the CR/PR group and was 482 days in the SD/PD group (Figure 5b).

### 3.5. Outcomes of Patients Undergoing Curative-Intent CIRT After Systemic Immunotherapy (ATZ + BEV or DUR + TRE)

**Figure 6:**
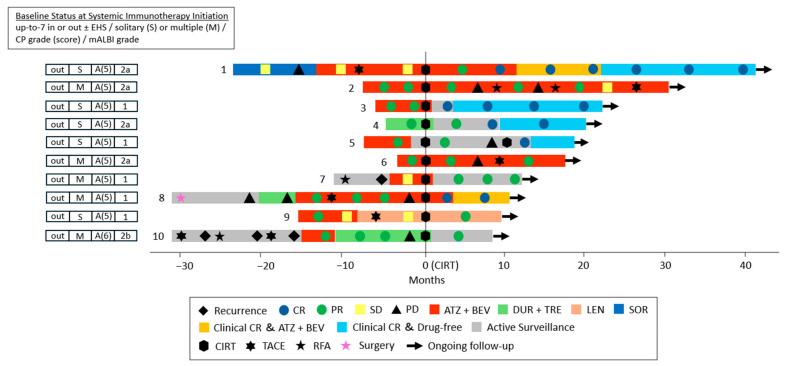
Swimmer plot illustrating treatment duration and best overall response in patients who underwent curative-intent CIRT after systemic immunotherapy (ATZ + BEV or DUR + TRE). ATZ + BEV, atezolizumab plus bevacizumab; DUR + TRE, durvalumab plus tremelimumab; LEN, lenvatinib; CAB, cabozantinib; CIRT, carbon-ion radiotherapy; EHS, extrahepatic spread; CR, complete response; PR, partial response; SD, stable disease; PD, progressive disease; TACE, transarterial chemoembolization; RFA, radiofrequency ablation; TAI, transarterial infusion.

Figure 6 presents a swimmer plot illustrating treatment duration and best overall response in 10 patients with multifocal intrahepatic HCC who initiated systemic immunotherapy—ATZ + BEV or DUR + TRE—and achieved downstaging, subsequently undergoing curative-intent CIRT. Among these 10 patients, 5 achieved clinical CR, and 4 achieved clinical CR and a drug-free status. Notably, all four patients who achieved clinical CR, followed by drug-free status, had a solitary intrahepatic tumor distribution pattern at the initiation of systemic immunotherapy.

Among the five patients who did not achieve clinical CR, three remained recurrence-free during the follow-up period, whereas the remaining two achieved disease control with additional locoregional therapies despite experiencing recurrence.

### 3.6. Outcomes of Patients Undergoing CIRT for Lesions with Insufficient Response (Including Oligolesions; ≤3 Lesions in Total) After Systemic Immunotherapy (ATZ + BEV or DUR + TRE)

**Figure 7:**
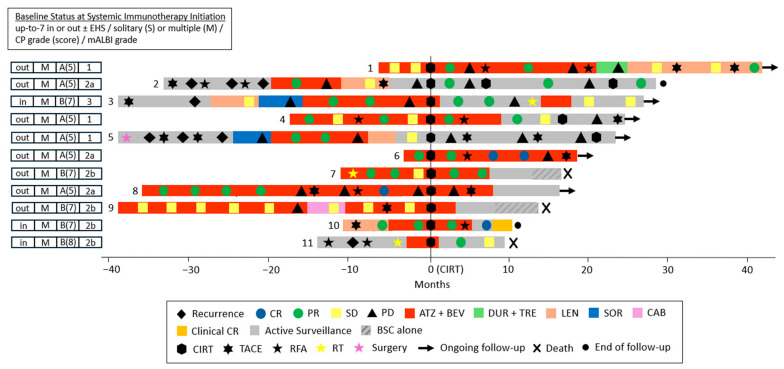
Treatment duration and best overall response. Swimmer plot showing treatment duration and best overall response in patients treated with CIRT for lesions with insufficient response, including oligolesions (up to 3 lesions), after systemic immunotherapy (ATZ + BEV or DUR + TRE). ATZ + BEV, atezolizumab plus bevacizumab; DUR + TRE, durvalumab plus tremelimumab; LEN, lenvatinib; SOR, sorafenib; CAB, cabozantinib; CIRT, carbon-ion radiotherapy; EHS, extrahepatic spread; CR, complete response; PR, partial response; SD, stable disease; PD, progressive disease; BSC, best supportive care; TACE, transarterial chemoembolization; RFA, radiofrequency ablation; RT, radiotherapy; TAI, transarterial infusion.

CIRT was administered to 11 patients with multifocal intrahepatic HCC who achieved tumor shrinkage to ≤3 lesions, including oligolesions, following systemic immunotherapy (ATZ + BEV or DUR + TRE). These patients were treated with CIRT for their bulky lesions and additional local therapies for the remaining oligolesions, with the aim of achieving a curative-intent treatment strategy. Figure 7 displays a swimmer plot showing treatment duration and the best overall response in these patients.

Among the patients treated with CIRT for bulky lesions, only three (Case 4, 6, 10) were able to receive additional local therapies for the remaining oligolesions; all additional local treatments consisted of radiofrequency ablation (Figure 7). Of the remaining 8 patients, 4 patients were unable to undergo additional local therapies due to deterioration in liver function, while the other 4 experienced disease progression before planned additional local treatments, leading to discontinuation of local therapy (Figure 7).

Among these 11 patients, only one patient achieved clinical CR, indicating a low rate of transition to clinical CR. In several patients, deterioration of liver reserve at the time of CIRT limited the feasibility of administering long-term systemic immunotherapy thereafter. Furthermore, even among patients who were able to continue systemic immunotherapy, recurrent lesions were observed in some cases, and adequate disease control could not be achieved in several patients.

### 3.7. Safety

Treatment-related AEs are summarized in Table 2. Among CIRT-related AEs, elevations of AST, ALT, and total bilirubin were observed in 6 patients (14.6%), 5 patients (12.2%), and 4 patients (9.8%), respectively. Grade ≥3 CIRT-related AEs included total bilirubin elevation in two patients (4.9%) and gastrointestinal bleeding in one patient (2.4%). Among systemic immunotherapy-related AEs, proteinuria was the most frequent (8 patients, 19.5%), followed by hypertension (6 patients, 14.6%). Grade ≥3 systemic immunotherapy-related AEs included proteinuria (9.8%), endocrinopathy (4.9%), pneumonitis (2.4%), and bevacizumab-related bleeding (2.4%).

Among the clinically significant events observed during sequential treatment, three patients (7.3%) discontinued systemic immunotherapy because of deterioration of liver function.

### 3.8. Outcomes of CIRT Monotherapy

For descriptive comparison, the baseline characteristics and clinical outcomes of patients who underwent CIRT monotherapy at our institution are presented in Supplementary Tables S1 and S2. Among 80 patients treated with CIRT monotherapy, the 1-, 2-, and 3-year OS rates were 93.1%, 91.1%, and 87.0%, respectively, while the corresponding PFS rates were 76.2%, 71.1%, and 64.7%. The 1-, 2-, and 3-year local control rates were 93.0%, 91.1%, and 80.8%, respectively. Formal statistical comparisons with the present study cohort were not performed due to differences in patient characteristics and treatment indications.

## 4. Discussion

This study yielded several novel findings. First, among 20 patients who received systemic immunotherapy with ATZ + BEV or DUR + TRE for recurrent HCC after CIRT, 14 achieved CR or PR, corresponding to an ORR of 70% and a DCR of 90%. Second, among patients treated with systemic immunotherapy for recurrent HCC after CIRT, most cases achieving CR/PR had tumor burden within the up-to-7 criteria and preserved liver function (mALBI grade 1 or 2a) at the initiation of systemic immunotherapy. In contrast, SD/PD cases were more frequently characterized by tumor burden exceeding the up-to-7 criteria or by extrahepatic spread at the initiation of systemic immunotherapy. Third, among patients who underwent curative-intent CIRT after systemic immunotherapy, a subset achieved clinical CR and were able to maintain a drug-free status; these patients uniformly demonstrated solitary intrahepatic tumor distribution at the initiation of systemic immunotherapy. Conversely, in patients who underwent CIRT for lesions with insufficient response (including oligolesions; ≤3 lesions in total) after systemic immunotherapy, the rate of transition to clinical CR was low, and many did not achieve adequate disease control.

**Figure 8:**
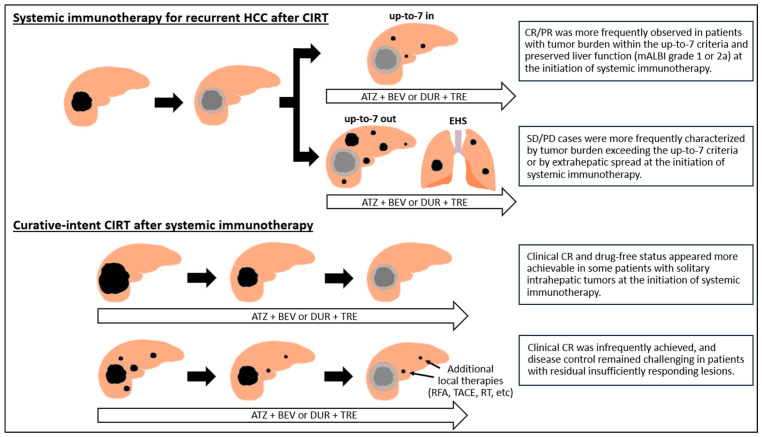
Hypothesis-generating clinical pathways for the integration of CIRT with systemic immunotherapy in patients with HCC. ATZ + BEV, atezolizumab plus bevacizumab; DUR + TRE, durvalumab plus tremelimumab; CIRT, carbon-ion radiotherapy; CR, complete response; PR, partial response; SD, stable disease; PD, progressive disease; mALBI, modified albumin–bilirubin; EHS, extrahepatic spread; RFA, radiofrequency ablation; TACE, transarterial chemoembolization; RT, radiotherapy.

Based on these findings, we propose hypothesis-generating clinical pathways for integrating CIRT and systemic immunotherapy in patients with HCC (Figure 8). These pathways outline the potential clinical scenarios suggested by the present study and are intended to guide future clinical investigation rather than serve as definitive treatment recommendations.

Particle beam radiotherapy represents one of the established locoregional treatment modalities for HCC, encompassing both CIRT and proton beam therapy. CIRT is distinguished by its high linear energy transfer and elevated relative biological effectiveness [15]. Furthermore, the Bragg peak effect allows CIRT to deliver highly precise doses to tumor sites, thereby achieving excellent local control while minimizing irradiation of the surrounding normal liver parenchyma [16]. The reduced number of treatment fractions further lessens the therapeutic burden and may contribute to preservation of quality of life, representing an additional clinical advantage of CIRT [16]. Outcomes of CIRT monotherapy at our institution (Supplementary Tables S1 and S2) were broadly consistent with previously reported findings [13,17,18], demonstrating excellent local control and favorable survival. Although direct comparisons must be interpreted cautiously due to differences in patient populations and treatment indications, these data provide a useful descriptive benchmark for contextualizing the present study.

In recent years, systemic immunotherapy has emerged as the first-line treatment for advanced HCC. Multidisciplinary strategies combining systemic immunotherapy with locoregional therapies have been proposed to achieve cancer-free or drug-free status in patients with Barcelona Clinic Liver Cancer stage B or C disease [19,20,21]. However, evidence regarding the efficacy of systemic immunotherapy in patients with recurrent HCC following CIRT, as well as data supporting treatment strategies that incorporate CIRT after systemic immunotherapy, remains extremely limited.

In this study, the efficacy of systemic immunotherapy for recurrent HCC after CIRT appeared to depend on intrahepatic and extrahepatic tumor distribution at recurrence. Specifically, patients whose tumor burden met the up-to-7 criteria at recurrence were more responsive to systemic immunotherapy and demonstrated significantly improved PFS and OS. The up-to-7 criteria are widely used as indicators of tumor burden in intermediate-stage HCC. Since disease beyond the up-to-7 criteria suggests the limited efficacy of locoregional therapy alone (e.g., transarterial chemoembolization), multidisciplinary treatment strategies incorporating systemic therapy have increasingly been advocated in recent years [22]. Therefore, a lower tumor burden at recurrence (within the up-to-7 criteria) may have contributed to the favorable disease control observed in our cohort.

In addition, among patients with recurrent HCC after CIRT, those with preserved liver function, as assessed by mALBI grade, demonstrated higher responsiveness to systemic immunotherapy. Recent studies have shown that liver functional reserve, assessed using ALBI or mALBI grade, is an important factor for stratifying clinical outcomes and prognosis in patients treated with ATZ + BEV [23]. Our finding that patients with mALBI grade 1/2a demonstrated favorable responses is consistent with these reports.

Furthermore, recent studies indicate that CIRT may induce immunogenic cell death and augment antitumor immunity when combined with immune checkpoint inhibitors [24]. Radiotherapy has also been shown to modulate the tumor microenvironment by facilitating tumor antigen release, enhancing dendritic-cell activation and CD8-positive T-cell infiltration, and inducing PD-1/PD-L1 signaling, thereby potentially increasing susceptibility to immune checkpoint blockade [25,26,27,28,29]. In addition, anti-VEGF therapy may promote vascular normalization [22] and improve immune-cell trafficking into the tumor microenvironment, which could further potentiate the immunomodulatory effects of CIRT [30]. Accordingly, although our clinical findings are consistent with the hypothesis of an immunological interaction between CIRT and systemic immunotherapy, the underlying biological mechanisms were not directly investigated and remain speculative.

Another important observation was the clinical relevance of CIRT administered after systemic immunotherapy. Among patients who achieved HCC downstaging with systemic immunotherapy and subsequently underwent curative-intent CIRT, a subset attained clinical CR or CR followed by drug-free status. Favorable outcomes appeared most evident in patients with solitary intrahepatic tumor distribution at the initiation of systemic immunotherapy. Previous studies have reported that, in patients treated with ATZ + BEV for HCC, cancer-free status is more likely in those with fewer intrahepatic lesions [31]. Conversely, curative conversion is challenging in patients with a high tumor burden [14]. These findings align with our observations.

By contrast, disease control appeared to be more challenging in patients who underwent CIRT for lesions with insufficient response (≤3 lesions in total) after systemic immunotherapy, with only a limited proportion ultimately achieving clinical CR.

Consequently, only a small number of patients proceeded to additional local therapies for oligolesions. Although recent reports have described regression of nonirradiated lesions—so-called abscopal effects—when radiotherapy is combined with ATZ + BEV [32,33], no clear evidence of such effects was observed in our cohort. Importantly, the sequential combination of CIRT and systemic immunotherapy demonstrated an acceptable safety profile. Most treatment-related AEs were manageable and consistent with the established safety profiles of CIRT and immune checkpoint inhibitor-based therapy. No unexpected toxicities attributable to the treatment sequence were observed, supporting the clinical feasibility of this multidisciplinary approach. Nevertheless, these findings should be interpreted cautiously given the small sample size and potential influence of treatment selection factors.

This study has several limitations. The small sample size and potential confounding factors—including tumor burden, liver function, performance status, and physician judgment—necessitate cautious interpretation. These findings should therefore be regarded as hypothesis-generating and require validation in larger multicenter studies. Moreover, comparisons among the three treatment groups should be interpreted carefully, as they represent distinct clinical scenarios rather than directly comparable cohorts. The limited number of patients in each subgroup precludes robust comparative analyses, reinforcing the descriptive and hypothesis-generating nature of the findings rather than evidence of superiority for any particular treatment sequence. Finally, although several clinical studies have suggested that radiotherapy may enhance antitumor immunity through immunogenic cell death and modulation of the tumor microenvironment, our study did not include mechanistic assessments such as immune profiling or biomarker analyses. Thus, any potential biological interaction between CIRT and systemic immunotherapy should be considered hypothesis-generating rather than established.

Nevertheless, a major significance of this study is the systematic evaluation of the efficacy of systemic immunotherapy in patients with recurrent HCC after CIRT, as well as the detailed assessment of clinical courses in patients who underwent additional CIRT after initiation of systemic immunotherapy.

## 5. Conclusions

In conclusion, systemic immunotherapy with ATZ + BEV or DUR + TRE demonstrated high efficacy in patients with recurrent HCC after CIRT, particularly among those within the up-to-7 criteria and preserved liver function (mALBI grade 1/2a) at recurrence. Furthermore, among patients who underwent curative-intent CIRT after systemic immunotherapy, a substantial proportion of those with a solitary intrahepatic tumor distribution at the initiation of systemic immunotherapy achieved clinical CR and remained drug-free. Conversely, among patients who underwent CIRT for lesions with insufficient response (including oligolesions; ≤3 lesions in total) to systemic immunotherapy, the rate of transition to clinical CR was low, and many did not achieve adequate disease control.

## Figures and Tables

**Table 1:** Comparison of Response and Disease Control Patterns in Patients with Recurrent HCC Post-CIRT Undergoing Systemic Immunotherapy.

Variable	CR/PR (*n* = 14)	SD/PD (*n* = 6)	*p*-Value
Sex (male/female)	10/4	4/2	>0.9999
Age (years)	77.5 (71.8–84.3)	78.5 (71.5–85.3)	0.5773
BCLC stage (A/B/C)	7/3/4	3/2/1	>0.9999
<At the time of CIRT>			
Maximum tumor size (mm)	59 (49–82.3)	53.5 (40–105)	0.9858
Child–Pugh score (5/6/7)	11/2/1	6/0/0	>0.9999
mALBI grade (1/2a/2b)	9/3/2	4/2/0	>0.9999
Up-to-7 criteria (in/out)	8/6	3/3	>0.9999
Macrovascular invasion (yes/no)	4/10	1/5	>0.9999
Extrahepatic spread (yes/no)	0/14	0/6	>0.9999
AFP (ng/mL)	182.1 (13.8–3510)	328.2 (4.0–1204)	0.1531
DCP (mAU/mL)	622 (37–2611)	316.5 (26–6556)	0.8117
<At initiation of systemic immunotherapy>			
Child–Pugh score (5/6/7)	10/4/0	2/3/1	0.1815
mALBI grade (1/2a/2b)	4/7/3	0/0/6	0.0076
Up-to-7 criteria (in/out)	10/4	3/3	0.6126
Macrovascular invasion (yes/no)	1/13	0/6	>0.9999
Extrahepatic spread (yes/no)	0/14	3/3	0.0175
AFP (ng/mL)	17.4 (6.4–535.5)	95.8 (2.9–20,419)	0.3917
DCP (mAU/mL)	85 (27–692)	692 (42.8–8184)	0.8659
First-line systemic immunotherapy (ATZ + BEV/DUR + TRE)	12/2	5/1	>0.9999

**Table 2:** Treatment-related adverse events and clinically significant events during sequential treatment.

Event	Any Grade, *n* (%)	Grade ≥3, *n* (%)
<CIRT-related adverse events>		
AST elevation	6 (14.6)	0 (0)
ALT elevation	5 (12.2)	0 (0)
Total bilirubin elevation	4 (9.8)	2 (4.9)
Gastrointestinal bleeding	1 (2.4)	1 (2.4)
<Systemic immunotherapy-related adverse events>		
Immune-related hepatitis	1 (2.4)	0 (0)
Pneumonitis	1 (2.4)	1 (2.4)
Colitis	0 (0)	0 (0)
Endocrinopathy	3 (7.3)	2 (4.9)
Proteinuria	8 (19.5)	4 (9.8)
Hypertension	6 (14.6)	0 (0)
Bevacizumab-related bleeding	1 (2.4)	1 (2.4)

## Data Availability

The data supporting the findings of this study are available from the corresponding author (H.H.) upon reasonable request.

## Supplementary Materials

The following supporting information can be downloaded at: https://www.mdpi.com/article/10.3390/jcm15145449/s1, Table S1: Baseline characteristics of patients treated with CIRT monotherapy (*n* = 80); Table S2: Clinical outcomes after CIRT monotherapy.

## Abbreviations

The following abbreviations are used in this manuscript:

AFP	Alpha-fetoprotein
ATZ	Atezolizumab
BCLC	Barcelona Clinic Liver Cancer
BEV	Bevacizumab
CIRT	Carbon-ion radiotherapy
CR	Complete response
DCP	Des-γ-carboxy prothrombin
DUR	Durvalumab
HCC	Hepatocellular carcinoma
mALBI	Modified albumin–bilirubin
OS	Overall survival
PD	Progressive disease
PFS	Progression-free survival
PR	Partial response
SD	Stable disease
TRE	Tremelimumab
