# Supplementary material for: Real-World Outcomes of Combined Carbon-Ion Radiotherapy and Systemic Immunotherapy for Hepatocellular Carcinoma (Atezolizumab Plus Bevacizumab or Durvalumab Plus Tremelimumab): A Single-Center Retrospective Study
Source: J Clin Med. 2026 Jul 12;15(14):5449. doi: 10.3390/jcm15145449 (PMC13411702; doi:10.3390/jcm15145449)
Supplement: Supplementary file 1 [file jcm-15-05449-s001.zip › jcm-4329807-supplementary.pdf]

Supplementary Table S1. Baseline characteristics of patients treated with CIRT monotherapy (n = 80)

Outcome	Value
Median OS, months	NR
1-year OS	93.10%
2-year OS	91.10%
3-year OS	87.00%
Median PFS, months	NR
1-year PFS	76.20%
2-year PFS	71.10%
3-year PFS	64.70%
Median LC, months	NR
1-year LC	93.00%
2-year LC	91.10%
3-year LC	80.80%

CIRT = Carbon-ion radiotherapy

mALBI = modified albumin-bilirubin grade

BCLC = Barcelona Clinic Liver Cancer staging system

AFP = alpha-fetoprotein

DCP = des-γ-carboxy prothrombin

Supplementary Table S2. Clinical outcomes after CIRT monotherapy

Variable	Carbon-Ion Radiotherapy (*n* = 80)
Age (years)	Median: 81 (range: 56–97)
Sex (male/female) (%)	62 (77.5)/18 (22.5)
mALBI grade (1/2a/2b/3) (%)	36 (45)/19 (23.8)/21 (26.3)/4 (5)
BCLC stage (A/B/C) (%)	66 (82.5)/3 (3.8)/11 (13.8)
Tumor size (mm)	Median: 51 (range: 10–142)
Number of tumors (1/2) (%)	77 (96.3)/3 (3.8)
Macrovascular invasion (yes/no) (%)	11 (13.8)/69 (86.3)
AFP (ng/mL)	4.95 (2–20,000)
DCP (mAU/mL)	146.5 (6.7–81,886)

Abbreviations

CIRT = carbon-ion radiotherapy

OS = overall survival

PFS = progression-free survival

LC = local control

NR = not reached
